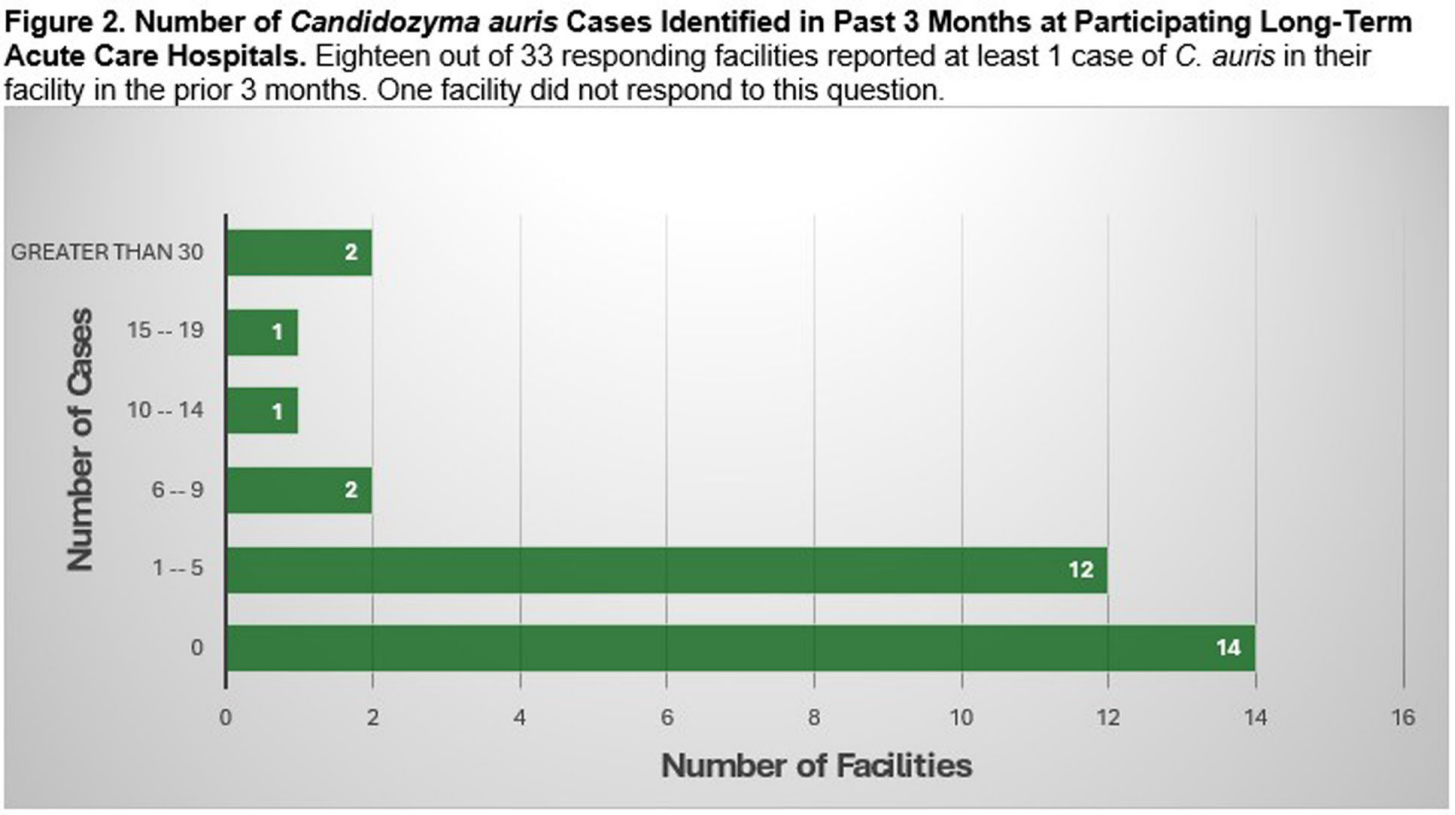# 280 IV to PO Antibiotic Utilization Dashboard: A Clinical Informatics Tool to Optimize Therapy Transitions

**DOI:** 10.1017/ash.2026.10641

**Published:** 2026-06-23

**Authors:** Kim Tran, Mckenzi King, Nidhi Undevia, Lahari Thotapalli, Hannakate Lichota, Lisa Duffner, Alison Peterson, Rachel Medernach, Ronda Cochran, Mary Carl Froilan, Aleksandra Kozicka, Mary Hayden, Sarah Sansom

**Affiliations:** 1 Rush University Medical Center; 2 RML Specialty Hospital; 3 Rush University

## Abstract

**Background:** We queried long term acute care hospitals (LTACHs) within the NALTH network regarding facility characteristics, C. auris prevention practices, and perceived barriers to C. auris prevention. **Methods:** Participating facilities completed a REDCap survey of multiple choice, free response, and ranked choice questions. Quantitative and descriptive analyses were performed for completed surveys. Perceived barriers and important tools for C. auris prevention were assessed with ranked choice questions and analyzed by non-weighted counts. **Results:** Among 33 responses from 56 eligible LTACHs (Figure 1, Table 1), 23 (70%) had ever identified at least one case of C. auris infection or colonization. Eighteen of 33 (54%) reported identifying cases of C. auris within the past 3 months (Figure 2) and 9 of 33 (27%) had experienced suspected within-facility transmission. All facilities employed multimodal approaches for C. auris infection prevention (Table 2). More than half of LTACHs reported routine C. auris screening programs (21, 64%), including in response to a known C. auris case (n=15) and/or screening for asymptomatic colonization at the time of LTACH admission (n=12). The most frequently reported barriers to effective C. auris infection prevention included lack of communication between healthcare facilities at the time of patient transfer (n=16), lack of training and education for frontline staff (n=15), and lack of compliance with personal protective equipment (n=12). The most impactful methods anticipated to support future C. auris prevention included improved communication between facilities at the time of patient transfer (n=15), standardized protocols for C. auris decolonization (n=15), and standardized protocols for C. auris screening and isolation (n=14). **Conclusion:** Most participating LTACHs within the NALTH network reported firsthand experience with C. auris. Universally, responding facilities employed multimodal evidence-based infection prevention and control methods targeted against C. auris. Future research should focus on improving inter-facility communication of C. auris colonization or infection status, development of novel decolonization strategies, and standardization of surveillance practices.

**Figure:**
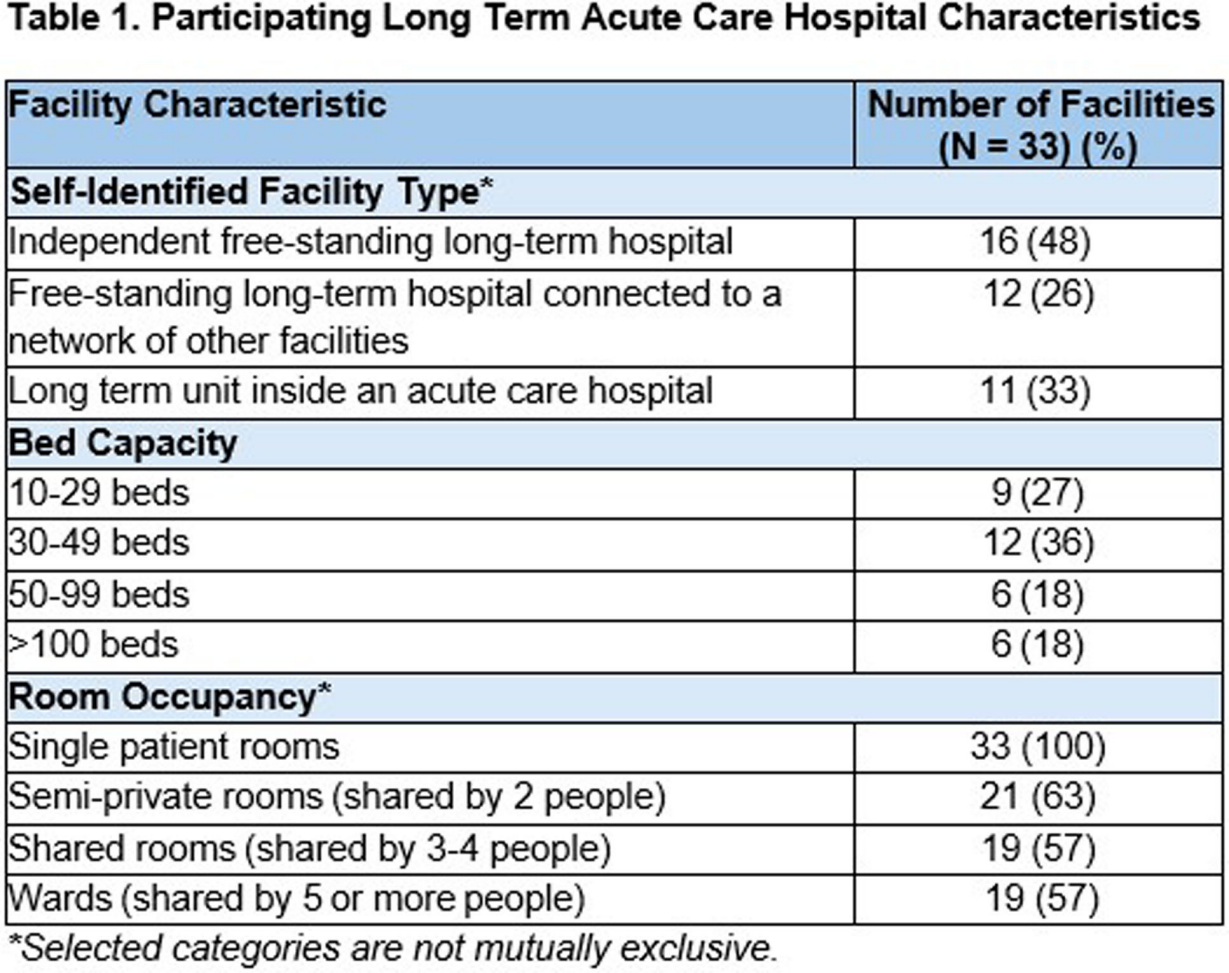

**Figure:**
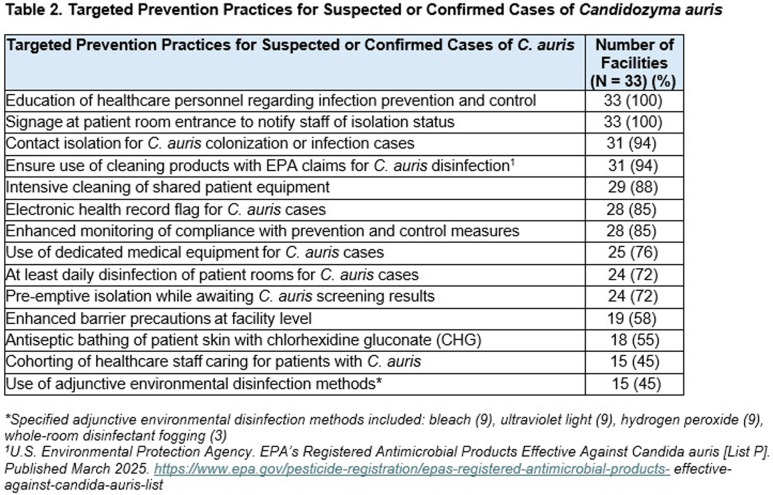

**Figure:**
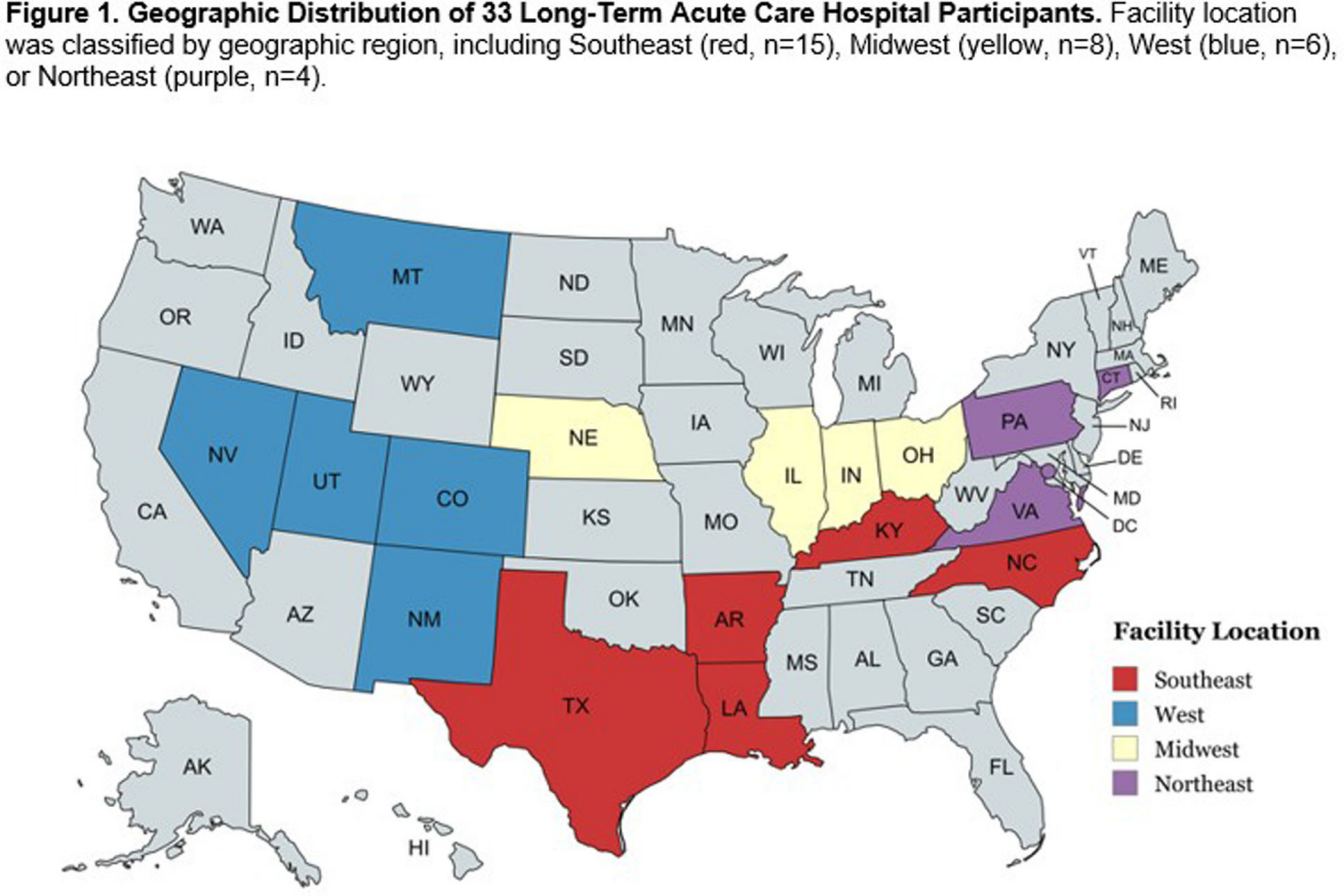

**Figure:**